# CRISPR/dCas9-mediated transcriptional improvement of the biosynthetic gene cluster for the epothilone production in *Myxococcus xanthus*

**DOI:** 10.1186/s12934-018-0867-1

**Published:** 2018-01-29

**Authors:** Ran Peng, Ye Wang, Wan-wan Feng, Xin-jing Yue, Jiang-he Chen, Xiao-zhuang Hu, Zhi-feng Li, Duo-hong Sheng, You-ming Zhang, Yue-zhong Li

**Affiliations:** 1 State Key Laboratory of Microbial Technology, School of Life Science, Shandong University, Jinan, 250100 China; 2 State Key Laboratory of Bioactive Substances and Functions of Natural Medicines, Institute of Materia Medica, Chinese Academy of Medical Sciences, Beijing, 100050 China

**Keywords:** CRISPR/dCas9 activation, sgRNAs, Activator proteins, Promoter, Transcriptional improvement, Biosynthetic gene cluster, Epothilones, *Myxococcus xanthus*

## Abstract

**Background:**

The CRISPR/dCas9 system is a powerful tool to activate the transcription of target genes in eukaryotic or prokaryotic cells, but lacks assays in complex conditions, such as the biosynthesis of secondary metabolites.

**Results:**

In this study, to improve the transcription of the heterologously expressed biosynthetic genes for the production of epothilones, we established the CRISPR/dCas9-mediated activation technique in *Myxococcus xanthus* and analyzed some key factors involving in the CRISPR/dCas9 activation. We firstly optimized the *cas9* codon to fit the *M. xanthus* cells, mutated the gene to inactivate the nuclease activity, and constructed the dCas9-activator system in an epothilone producer. We compared the improvement efficiency of different sgRNAs on the production of epothilones and the expression of the biosynthetic genes. We also compared the improvement effects of different activator proteins, the ω and α subunits of RNA polymerase, and the sigma factors σ54 and CarQ. By using a copper-inducible promoter, we determined that higher expressions of dCas9-activator improved the activation effects.

**Conclusions:**

Our results showed that the CRISPR/dCas-mediated transcription activation is a simple and broadly applicable technique to improve the transcriptional efficiency for the production of secondary metabolites in microorganisms. This is the first time to construct the CRISPR/dCas9 activation system in myxobacteria and the first time to assay the CRISPR/dCas9 activations for the biosynthesis of microbial secondary metabolites.

**Electronic supplementary material:**

The online version of this article (10.1186/s12934-018-0867-1) contains supplementary material, which is available to authorized users.

## Background

Clustered regularly interspaced short palindromic repeats (CRISPR) and the CRISPR associated (Cas) nuclease are widely distributed in bacteria and archaea, playing as an RNA-mediated adaptive immune system to cleave the invasive DNA [1, 2]. When the nuclease activity of Cas9 is inactivated, the nuclease-dead protein (dCas9) is able to bind to the target sequence site with no cleavage activity. If guided to a suitable site, the dCas9 fusion with a transcriptional activator domain is able to recruit RNA polymerase (RNAP) to activate and improve the transcription of the target genes [3–6]. Compared with the traditional expression techniques that are based on the modification of genes and promoters, the CRISPR/dCas-mediated transcription activation method (CRISPRa) is simple, target-specific, programmable and frequently applicable [4, 6, 7]. Researchers have successfully employed the CRISPRa system to dissect the limiting component of a biochemical process, identify the molecular target of a drug, or activate key rate-limiting steps in a pathway. For example, Bikard et al. reported the programmable transcription activation of *lacZ* and *gfp* reporter genes in *E. coli* through a fusion of the ω subunit of RNAP to the C-terminus of dCas9; and a maximum of 23-fold induction was achieved with its Protospacer Adjacent Motif (PAM) positioned at the 59-nt upstream of the − 35 element [3]. However, the CRISPR/dCas9 activation system has not yet applied in complex conditions, such as the biosynthesis of microbial secondary metabolites. The biosynthesis of secondary metabolites in microorganisms often requires a collaboration of multiple enzymes, which are encoded by many genes that are organized in a big gene cluster. Transcription of those genes is complexly regulated [8], and efficient improvement and activation of the transcription of a microbial secondary metabolism is a challenge.

Recently, myxobacteria have been found to be the top microbial producers of secondary metabolites like fungi, actinomycetes and some species of the genus *Bacillus* [9–11]. Due to limited genetic performing techniques, studies of transcriptional regulation in myxobacteria are hampered. There are only a few reports on the transcriptional regulation for the biosynthesis of secondary metabolites in myxobacteria [11, 12]. Epothilone is a kind of antitumor polyketides discovered in the broth of *Sorangium cellulosum* [13, 14], biosynthesized by a 56-kb gene cluster [15]. In our previous studies, we integrated the whole gene cluster, including the promoter sequence, from *S. cellulosum* So0157-2 [16] into the *M. xanthus* genome by transposition insertion [17]. In this study, we attempted to establish the CRISPR/dCas9-mediated activation technique in *M. xanthus*, and investigated its improvement efficiency on the transcription of the heterologously expressed biosynthetic gene cluster for the production of epothilones. We compared the improvement efficiencies of different sgRNAs, different activator proteins, and the dCas9-activator expression levels on the transcriptions of different genes in the big gene cluster and the production of epothilones in *M. xanthus*. Our results suggested that CRISPRa is a simple and broadly applicable technique to improve the transcriptional efficiency of microbial secondary metabolites.

## Results and discussion

### Codon-optimization of *cas9* to fit the *Myxococcus xanthus* cells

The GC-content of the *cas9* gene from *Streptococcus pyogenes* is 35.1%, which is much lower than the 68.9% percentage of *M. xanthus* DK1622 genome. We optimized the *cas9* codon according to the codon usage bias in *M. xanthus* by using the JCAT software, and named the optimized gene as *mxcas9* and the corresponding protein sequence was named as mxCas9. The sequence was deposited in GenBank under the ID number of 2074034. In addition to the codon optimization, we also removed most commonly used enzymatic sites in the gene to facilitate further genetic manipulation. After the optimization, the GC content of the gene reaches 61.4%, and the codon adaptation index (CAI) value of *mxcas9* has been adjusted from 0.05 to 0.97 for *M. xanthus*.

**Fig. 1:**
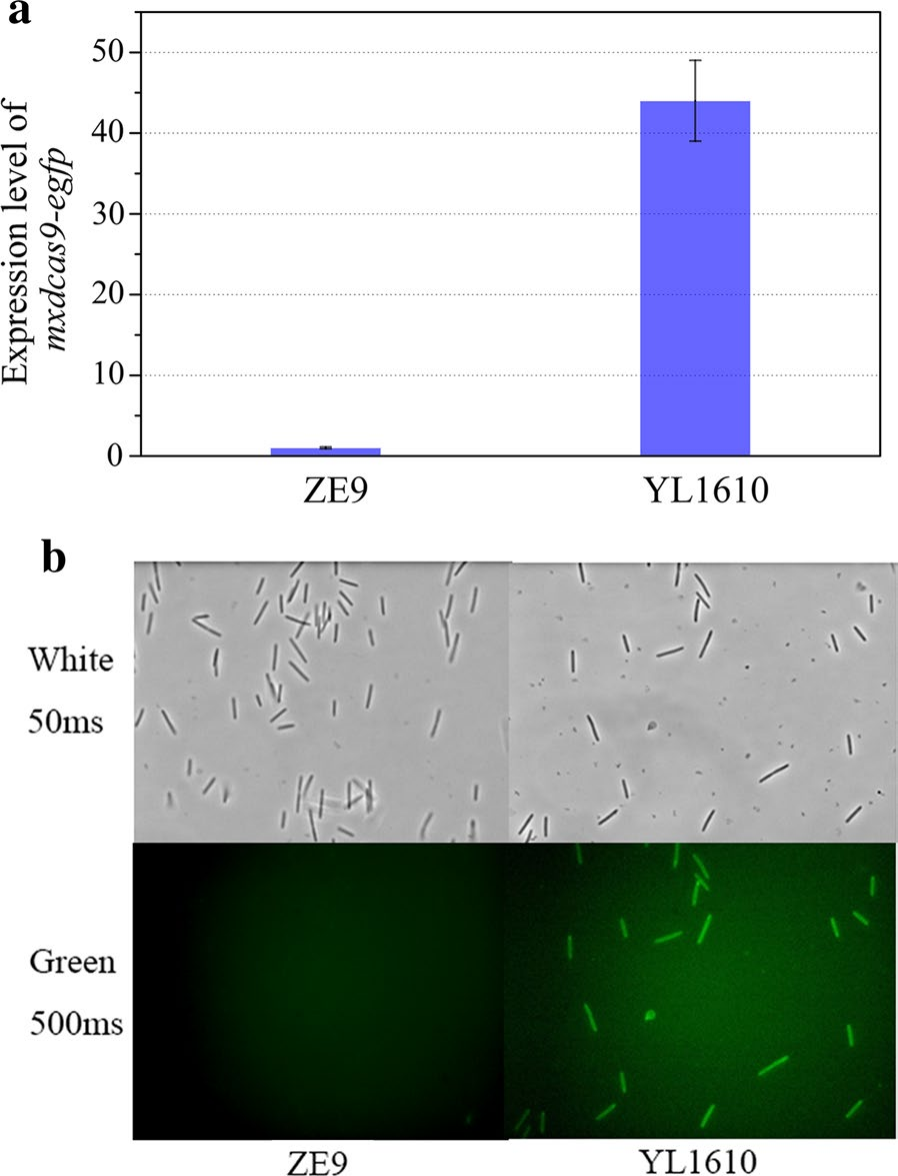
Expression of *mxdcas9* in mutant YL1610 (DK1622::pSWdmxCas9gfp) and the ZE9 control, measured by RT-qPCR amplification of the *dcas9* gene (**a**) or fluorescence under a fluorescence microscope with the 500 ms exposure time (**b**). In this experiment, the *M. xanthus* strain ZE9 was used as a control

To generate nuclease-dead Cas9 protein, we made substitutions in *mxcas9* (D10A and H840A; referred to Additional file 1: Figure S1) according to the studies by Jinek et al. [18], and named the gene *mxdcas9*. The gene was inserted into the pSWU30 plasmid under the control of T7A1 promoter [19, 20]. The constructed plasmid, named pSW30-mxdCas9, contains an Mx8 site for the insertion in the genome of *M. xanthus* (Additional file 2: Figure S2). To check the expression of *mxdcas9* in *M. xanthus*, the gene of fluorescence reporter EGFP was fused to the 3′-end of the *mxdcas9* gene to get a new plasmid named pSWdCas9gfp (Additional file 3: Figure S3). This plasmid was transformed by electroporation into *M. xanthus* DK1622. The RT-qPCR amplification result showed that the *mxdcas9*-*egfp* fused gene was able to express (Fig. 1a). We named this strain YL1610. Observed under the 500 ms exposure time, the *M. xanthus* cells transferred with the pSWdCas9gfp plasmid exhibited green fluorescence, while the cells of *M. xanthus* without the plasmid did not (Fig. 1b). The result indicated that the *mxdcas9* gene was able to express in *M. xanthus* cells normally.

### Effects of different sgRNA-binding sites on the improvement of epothilone production

**Fig. 2:**
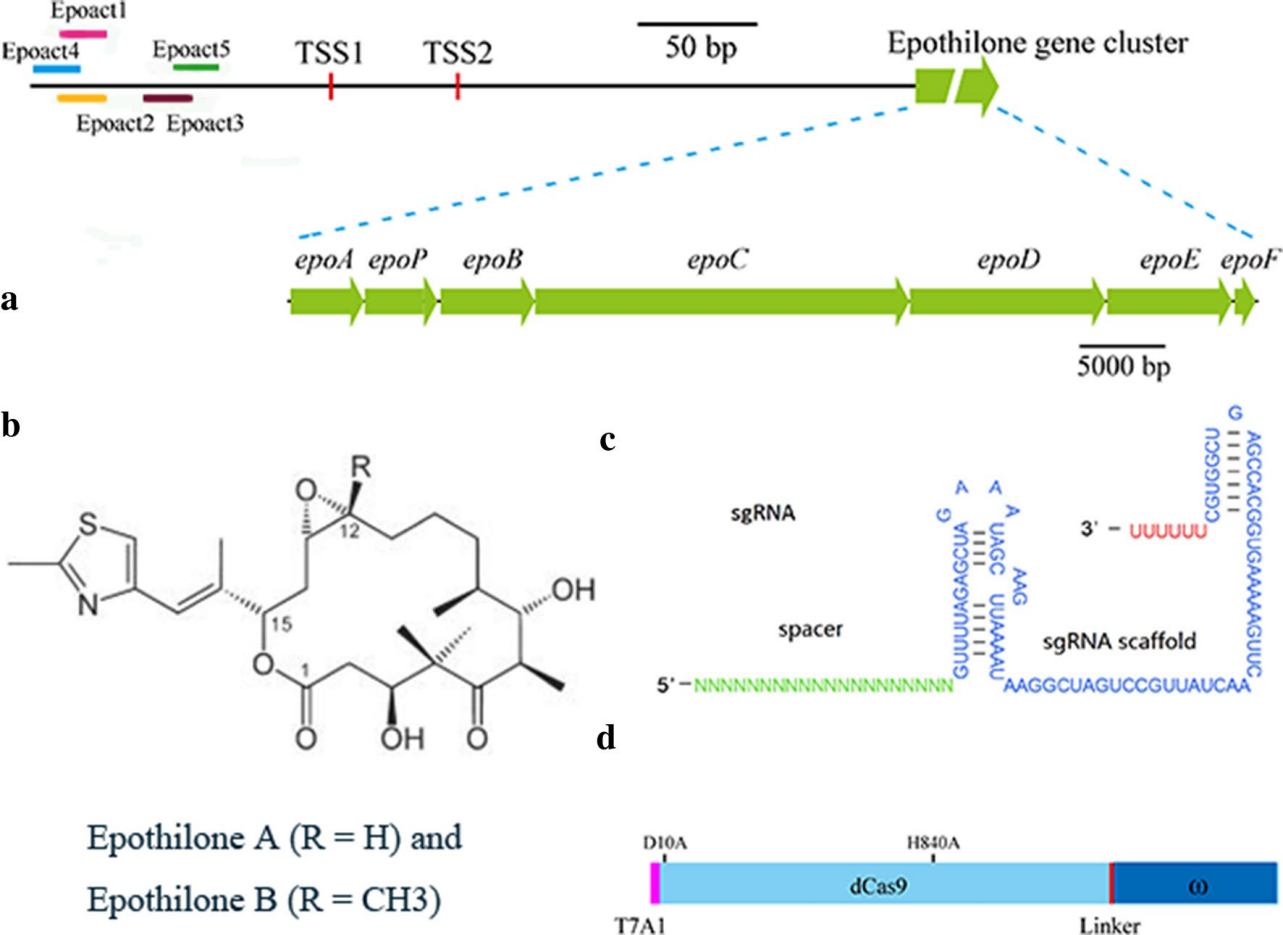
Design of the sgRNA and the mxdCas9-activator fusion for RNA-guided transcriptional activation. **a** A physical map of the promoter region for the epothilone biosynthetic gene cluster, showing the places of five designed spacers. The epothilone genes are shown in green arrow, and the two TSSs are shown with red lines. The different spacer sequences are shown in bars with different colors. **b** The molecular structures of epothilones A and B. **c** Design of sgRNA scalfold, containing a 20-nt specific targeting sequence, the tracrRNA scaffold and a U6 terminator. **d** The ω subunit protein was designed to fuse to the C-terminal of the mxdCas9 with a linker. This fusion gene is under the control of T7A1 promoter. The two mutation sites (D10A and H840A) are shown

Judged by their overlapping stop, the presence of the ribosome-binding site of the downstream gene within the coding sequence of the upstream gene and no apparent intergenic transcriptional terminator, the big gene cluster for the biosynthesis of epothilones was suggested to be transcriptionally coupled as an operon under the controlled by the promoter region immediately preceding *epoA* [15]. We previously determined that, by using the primer extension analysis technique, the 841 nucleotide (nt) promoter sequence of the biosynthetic gene cluster contains two transcriptional start sites (TSSs), one is at 246 bp (TSS1) and the other is at 193 bp (TSS2) in front of the translation start site (TLS) for the transcription [17]. TSS1 has strong promotion activity for transcription while the effect of TSS2 is weak [21]. Figure 2a sketches the gene cluster for the biosynthesis of epothilones and Fig. 2b exhibits the two major components of epothilones (epothilones A and B). In order to activate the whole epothilone gene cluster for the production of epothilones, we designed the spacer target sequences complemented on the promoter sequence (Fig. 2a). Single guide RNA (sgRNA) sequence, a combination of the CRISPR associated RNA (crRNA) and the trans-activation crRNA (tracrRNA), was constructed according to the previous method [18] for to guide mxdCas9 to the target DNA sites. We designed five spacers for the construction of sgRNAs, which contained a 20-nt specific target sequence, a tracrRNA scaffold and a U6 terminator (Fig. 2c). Bikard et al. once demonstrated that approximately 80–110-nt before a TSS provides an optimal distance for a RNA polymerase to bind to the promoter and to increase gene expression [3]. Because there are two TSSs in front of the TLS of the epothilone gene cluster, the five spacers were designed with a distance ranging from 53 bp (Epoact5) to 103 bp (Epoact4) upstream of TSS1 (Fig. 2a). In parallel, the distance range is 106 bp to 156 bp upstream of TSS2. The 20-nt spacers Epoact1, 4 and 5 are on the coding strand, while the Epoact2 and 3 are on the non-coding strand. Each CRISPR target sequence has an “NGG” PAM sequence at the 3′-end of the spacer for proper binding between the protein-RNA complex and the target DNA sequence. We used the *pilA* promoter from *M. xanthus* to drive the transcription of sgRNA. The off-target efficiency of spacer was calculated by using the 1.0 version of the software CasOT [22]. Because a spacer with high off-target efficiency does not function well in the transcriptional activation [23–25], four spacers (Epoact2, 3, 4 and 5) were designed having low off-target efficiency, and Epoact1, of which the off-target prediction was high, was employed as a control. Detailed information of these spacers and their distances to TSS1/TSS2 are listed in Table 1.

**Table 1:** The designed spacer sequences based on the promoter of epothilone gene cluster

Name	Spacer	Coding strand	Location	Off-target prediction^a^
Epoact1	TGGCCCTTTGAGGGGCTGGC	+	95 bp from TSS1 148 bp from TSS2	145
Epoact2	TCGCGCGATATCGTCGACCC	–	93 bp from TSS1 146 bp from TSS2	21
Epoact3	TTACCCTCGGGGAATTGACT	–	67 bp from TSS1 120 bp from TSS2	9
Epoact4	CATGAAAATGGCCCTTTGAG	+	103 bp from TSS1 156 bp from TSS2	20
Epoact5	TCTCCCAGTCAATTCCCCGA	+	53 bp from TSS1 106 bp from TSS2	18

^a^The potential off-target sites in the genome of *M. xanthus* DK1622

**Fig. 3:**
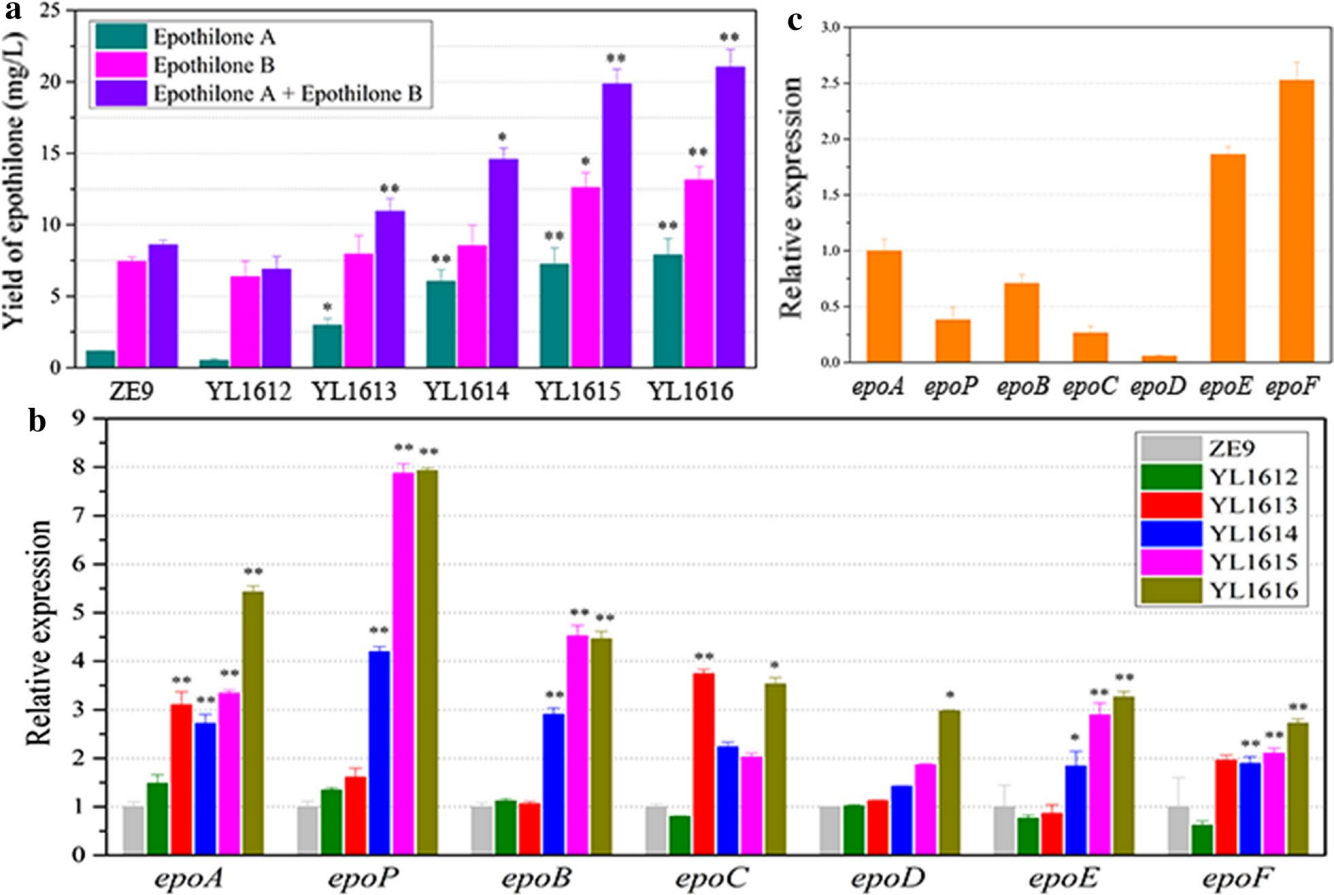
Activation effects of the epothilone gene cluster by the ω subunit of RNA polymerase. **a** The yields of epothilones A and B and their summation in mutants with the p41sg plasmids containing different spacers (YL1612, 95-nt TSS1/coding; YL1613, 93-nt TSS1/noncoding; YL1614, 120-nt TSS2/noncoding; YL1615, 103-nt TSS1/coding; YL1616, 106-nt TSS2/coding) and the ancestral strain *M. xanthus* ZE9. **b** RT-qPCR analysis of expression levels of the seven genes for the biosynthesis of epothilones in different mutants at 48 h of incubation. The expressions of the seven genes of the gene cluster for the biosynthesis of epothilones in *M. xanthus* ZE9 were each set as 1, and the expressions of the seven genes of mutant strains were shown as the relative expressions to that of their corresponding genes in ZE9. The error bars represent the standard deviation of three independent experiments. For statistical analysis between the ancestral strain and mutant strains, the signals of ** and * mean *p* < 0.01 and *p* < 0.05, respectively. **c** RT-qPCR analysis of the expression levels of the seven genes for the biosynthesis of epothilones of *M. xanthus* ZE9 at 48 h of incubation. The expression of *epoA* gene was set as 1, and the expressions of the other six genes in ZE9 were the relative expressions to that of *epoA*

Bikard et al. demonstrated that the fusion of dCas9 and ω protein could increase the transcription level of the targeted gene and corresponding β-galactosidase activity in either *E. coli* or *S. pneumoniae* [3]. We fused the ω protein gene from *M. xanthus* DK1622 (Table 2) to the 3′-end of *mxdcas9* with a 27-nt linker (Fig. 2d), and constructed pSWdCas9-ω by cloning the fused gene into the pSW30 plasmid. The plasmid was transformed by electroporation into *M. xanthus* ZE9 to obtain the mutant strain YL1611. We inserted different sgRNA sequences, each containing one of the five selected spacers Epoact1–Epoact5, into the autonomously replicative plasmid pZJY41, respectively (the constructed plasmids were named as p41sg1 to p41sg5). These plasmids were then transformed by electroporation into YL1611, respectively, obtaining the YL1612–YL1616 mutant strains (Additional file 4: Figure S4). We found that, except the Epoact1-containing YL1612, the other four mutant strains showed a significant increase in the production of either epothilone A or epothilone B (Fig. 3a). The production of epothilones A and B were 6.08- and 1.70-fold higher in YL1615, or 6.80-fold and 1.77-fold higher in YL1616 than that in ZE9, respectively. The distances between the binding site and TSS1/TSS2 are either 103 bp/156 bp for Epoact4 (in mutant YL1615), or 53 bp/106 bp for Epoact5 (in mutant YL1616). Since an optimal space for RNA polymerase binding to the promoter is approximately 80–110-nt before a TSS [3], we suggested that the production increase in YL1615 was probably majorly due to the increased RNA polymerase at the TSS1 site, while the increase in YL1616 majorly resulted from the increased enzyme at the TSS2 site. Thus, the recruiting efficiency of different sgRNAs was probably similar for either the TSS1 or TSS2 sites. The results from YL1613 (Epoact2) and YL1614 (Epoact3), in which the Epoact2 and Epoact3 spacers were designed on the non-coding strand, showed an agreement with the suggestion, i.e. sgRNAs having similar recruiting efficiency for either the TSS1 or TSS2 sites. Compared with to the ancestral strain ZE9, the epothilone production did not increase in YL1612. We suggested that the high off-target efficiency of Epoact1 was probably the reason for the inability of Epoact1 in activating the biosynthesis of epothilones.

**Table 2:** Information of the four activators

Name	Gene	Location	Amino acid	Function
ω subunit	*MXAN_4890*	6123084..6123320	78	DNA-directed RNA polymerase subunit omega
α subunit	*MXAN_3326*	3869058..3870080	340	DNA-directed RNA polymerase subunit alpha
CarQ	*MXAN_4088*	5034964..5035515	183	RNA polymerase sigma factor
σ54	*MXAN_1061*	1238621..1240138	505	RNA polymerase sigma-54 factor

The assembling line for the production of epothilones is a hybrid complex of PKS/NRPS enzymes. The length of the epothilone gene cluster is approximately 56-kb, containing seven unidirectional ORFs (referred to Fig. 2a), encoding five polyketide synthases (PKSs, including EpoA, EpoB, EpoC, EpoD, EpoE), a non-ribosomal peptide synthetase (NRPS, EpoP) and a cytochrome P450 oxidase (EpoF). Compared with the expressions in ZE9, the transcriptions of different genes increased significantly in these mutant strains, except YL1612 (Fig. 3b). Interestingly, the transcriptional levels of the first three genes increased more, while the transcription improvements of the hinder genes were less. For example, compared with that of ZE9, the transcriptional increases of the first three genes, i.e. *epoA*, *epoP* and *epoB* in YL1614 and YL1616 were 2.72-/4.19-/2.90-fold and 5.43-/7.93-/4.46-fold, respectively; whereas the increases of the following *epoC*, *epoD*, *epoE* and *epoF* genes in these two strains were 2.23-/1.42-/1.83-/1.89-fold and 3.52-/2.97-/3.26-/2.71-fold, respectively. The above results suggested that the fusion of mxdCas9 and the ω protein was able to be guided by the sgRNA sequence bound onto the promoter upstream sequence, and then recruited more RNAP proteins to improve the transcription of the epothilone gene cluster. The improvement was efficient for the whole gene cluster.

Notably, although the gene cluster was suggested to be co-transcribed under the control of the promoter preceding *epoA* [15] and the mxdCas9-activator was able to improve the transcription of the whole epothilone gene cluster, the seven genes significantly varied their expressions (Fig. 3c), which is similar to the transcriptomics analysis result of *M. xanthus* recombinants with the insertions of epothilone biosynthetic genes [17]. We presumed that there were some possible additional internal promoters within the gene cluster, which might involve in the transcriptional regulation of the clustered genes. The biosynthetic gene cluster has to be rechecked and further studies are required to be made on the possible internal promoters.

Another interesting result was that, while the productions of epothilones A and B were both significantly increased in YL1615 and YL1616 (containing Epoact4 and Epoact5, respectively), the Epoact2 and Epoact3 spacers in YL1613 and YL1614 increased the production of epothilone A significantly only (2.57- and 5.21-fold higher than that of ZE9, respectively) but not that of epothilone B (Fig. 3a). It is known that epothilones are normally produced as a mixture of epothilones A and B (two major components) in either the original *S. cellulosum* producers [13, 26] or the heterologous *M. xanthus* hosts [17, 27]. The difference between epothilones A and B is the presence of a methyl group at carbon 12 of epothilone B (referred to Fig. 2b). The biosynthesis of mixed epothilones A and B was suggested to be due to the promiscuity in the selection of malonyl-CoA and methylmalonyl-CoA by the second acyltransferase of the EpoC [15, 28, 29]. Thus, the availability of malonyl-CoA and methylmalonyl-CoA will affect the ratio of epothilones A and B produced [27]. We presumed that the unilateral significant increase of epothilone A in the YL1613 and YL1614 strains was probably due to the side effects of Epoact2 and Epoact3 spacers on the alteration of malonyl-CoA and methylmalonyl-CoA pools, which, however, requires detailed studies.

### Effects of different activation factors on the transcriptional improvement and epothilone production

Dove et al. illustrated that the combination of a DNA-bound protein (lambda cI repressor) and any RNAP component can improve the RNAP transcription efficiency in *E. coli* cells [30] In addition to the ω-subunit of RNAP, we further assayed activation effects of different transcriptional factors, including the α-subunit of RNAP, sigma factors σ54 and CarQ. Sigma factors control the specificity of gene transcription by binding to RNAP and the fusion of a sigma factor to dCas9 was probably able to recruit more RNAP to the promoter sequence. As the specific sigma factor for epothilone biosynthesis was unknown yet, we chose the global sigma factor σ54 and the ECF (extracytoplasmic function) sigma factor CarQ for the assay. CarQ is known to be responsible for the transcription initiation of *carQRS*, the central genes for carotenogenesis in *M. xanthus* [31]. We presumed that any activator protein that was fused to dCas9 was probably able to recruit more RNA polymerases to activate and improve the transcription of the target genes when guided by a specific sgRNA to a suitable site. The detailed information of these four activator proteins is shown in Table 2. We fused α subunit, σ54 and CarQ respectively to the dCas9-activator by using the similar process for the ω subunit, and constructed plasmids pSWdCas9-α, pSWdCas9-σ54 and pSWdCas9-CarQ using the similar process for the construction of pSWdCas9-ω (Additional file 3: Figure S3). These plasmids were transformed by electroporation into *M. xanthus* ZE9 to obtain mutant strains YL1617, YL1618 and YL1619, respectively. We transformed the pZJY41 plasmid with the Epoact5-containing sgRNA sequence into these three mutants, obtaining the mutant strains YL1620, YL1621 and YL1622, respectively.

**Fig. 4:**
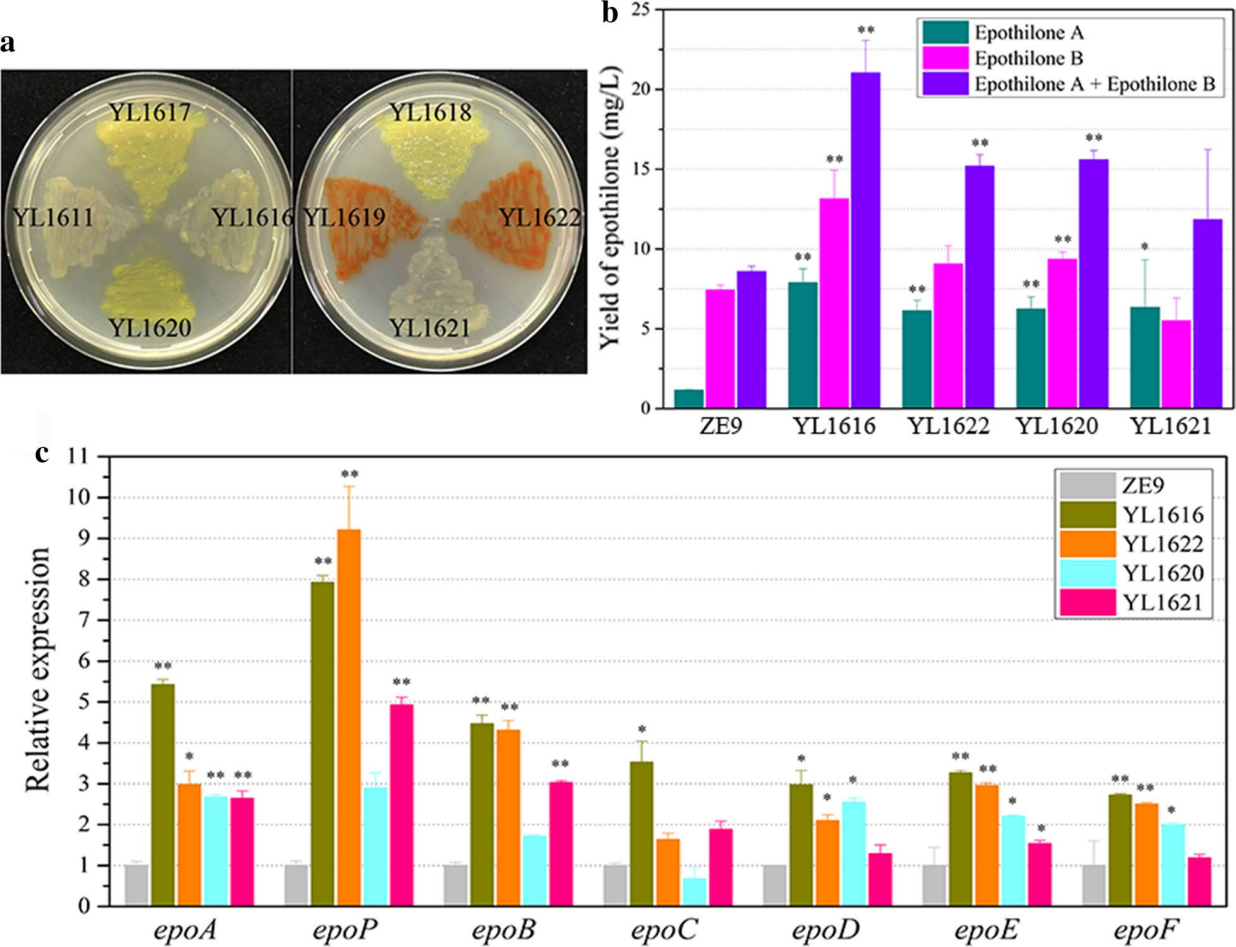
Effects of different activation proteins (ω, α, σ54, CarQ) on the growth (**a**), epothilone production (**b**) and the gene expression (**c**) of different *M. xanthus* mutants (**a**). The growth of different *M. xanthus* mutants on CYE plate. Mutants: YL1611 (ZE9::pSWdmxCas9-ω), YL1616 (ZE9::pSWmxdCas9-ω-p41sg5), YL1617 (ZE9::pSWmxdCas9-α), YL1618 (ZE9::pSWmxdCas9-σ54), YL1619 (ZE9::pSWmxdCas9-CarQ), YL1620 (ZE9::pSWmxdCas9-α-p41sg5), YL1621 (ZE9::pSWmxdCas9-σ54-p41sg5), YL1622 (ZE9::pSWmxdCas9-CarQ- p41sg5). **b** The yield and the summation of epothilones A and B in mutants (YL1616, YL1620, YL1621, YL1622) and *M. xanthus* ZE9. **c** RT-qPCR analysis of the seven genes for the biosynthesis of epothilones in *M. xanthus* strains at 48 h of incubation. The expressions of the seven genes in ZE9 were each set as 1, and the expressions of seven genes in mutant strains were the relative expressions to them. The error bars represent the standard deviation of three independent experiments. For statistical analysis between the ancestral type strain and mutant strains, the signals of ** and * mean *p* < 0.01 and *p* < 0.05, respectively

The cultures of these mutant strains showed different colors on CYE plate, and the strains with the same introduced activators normally had the same colony colors (Fig. 4a). For example, the colonies of the YL1619 and YL1622 strains with pSWdCas9-CarQ were red. This is probably also a side effect of mxdCas9-CarQ on carotenogenesis, suggesting the CRISPR/dCas9-mediated activation is often not strand-specific [32]. However, the fermentation result showed that the three activator-proteins were also able to increase the production of epothilones significantly (Fig. 4b). The improvement efficiencies caused by different activators were similar in the production of epothilone A, but varied in epothilone B. The activation effects of the α-subunit and CarQ were similar, but both were weaker than the ω-subunit did. Among them, the global sigma factor σ54 showed the weakest effect for the production of epothilones. The results suggested that, besides the activation effects on the target epothilone biosynthesis, different activator proteins might have additional activation effects on other genes, and these unrequired additional activation effects were dependent upon the characteristics of the activators, as well as the off-target efficiency of spacers. The RT-qPCR analysis revealed that, consistent with the increase in production of epothilones, the expression levels of the seven ORFs in the epothilone biosynthetic gene cluster were mostly increased (Fig. 4c). Similarly, the transcriptional levels of the former genes were higher than that the hinder genes.

### Effects of the expression levels of dCas9-activator-fusion on the gene transcription and epothilone production

To investigate effects of the expression levels of mxdCas9-activator-fusion, we replaced the T7A1 constitutive promoter with an inducible promoter. In *M. xanthus*, four inducible expression systems have been developed, i.e. the light-inducible CarQ promoter [33–35], the IPTG-based P*pilA*-*lacO*-RBS promoter [36], the MerR like repressor CarH-based promoter [37, 38], and the copper-inducible promoter [39]. Among them, the copper-inducible promoter is suitable for the general usage for its simple and highly controllable manner that the *cuoA* promoter is inducible by the addition of copper and the transcription level by the promoter is linearly dependent upon the copper concentration within a suitable extent [39, 40]. There is almost no influence on the growth of *M. xanthus* cells with the addition of a copper concentration up to 500 μM in medium [39].

**Fig. 5:**
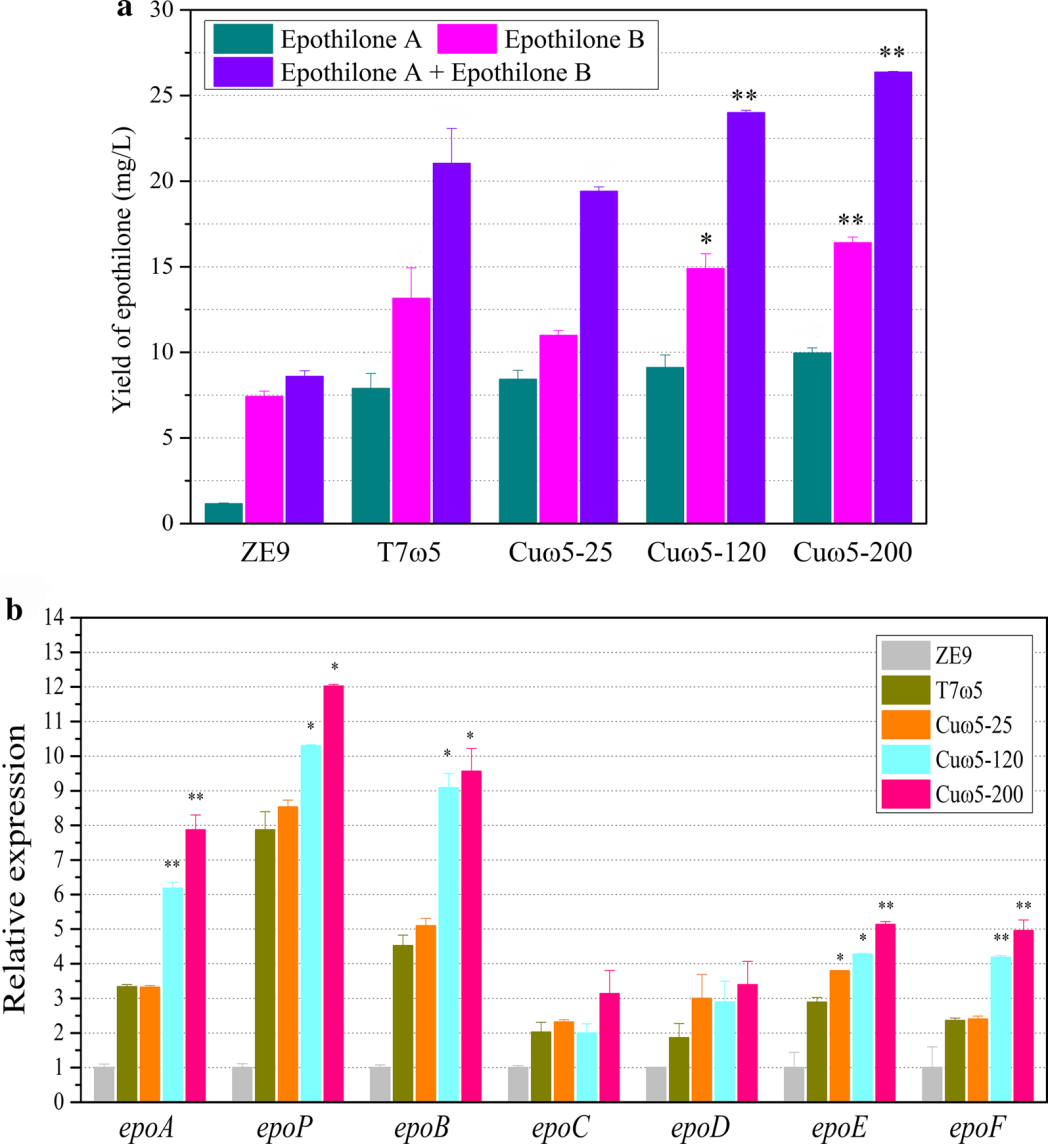
Activation of the epothilone gene cluster under the control of copper-inducible promoter. **a** The yield of epothilones A and B in mutants YL1616, YL1624 (25 μM CuSO_4_), YL1624 (120 μM), YL1624 (200 μM) and *M. xanthus* ZE9. **b** RT-qPCR analysis of the expression levels of seven genes of mutants YL1616, YL1624 (25 μM), YL1624 (120 μM), YL1624 (200 μM). The expressions of the seven genes in *M. xanthus* ZE9 at 48 h of incubation were set as 1, and the expressions of the seven genes of mutant strains were the relative expressions on them. The error bars represent the standard deviation of three independent experiments. For statistical analysis between wild type strain and mutant strains, ** and * mean *p* < 0.01 and *p* < 0.05, respectively

We chose the copper-inducible promoter *cuoA* to drive the expression of mxdCas9-ω subunit with the guidance of the Epoact5 sgRNA. First, we constructed plasmid pSWcuodCas9-ω and electroporated it into ZE9 to obtain mutant strain YL1623. Then, the p41sg5 plasmid was electroporated into YL1623, producing the mutant YL1624. In our previous studies on the CRISPR/Cas9–induced deletion, we demonstrated that the *M. xanthus* cells possessing the *cuoA* promoter-controlled *cas9* gene were unable to tolerate a high concentration of copper; the strain grew normally at 15 μM copper concentration and the 25 μM concentration was lethal for the strains [40]. However, when we cultivated the *M. xanthus* strain with the *cuoA*-induced *mxdcas9* gene in media containing 500 μM concentration of CuSO_4_, the cells showed almost the same growth ability as that of the strain with T7A1-controlled *mxdcas9* under the conditions without the addition of copper, which was probably due to the inactivation of the mxdCas9 nuclease activity. We compared the epothilone production abilities of the mutant strains YL1616 (the mxdCas9-ω fusion was driven by the constitutive promoter T7A1) and YL1624. The YL1616 strain was grown in normal cultures without the addition of copper, while the YL1624 strain was grown with the addition of 25, 120 and 200 μM concentration of CuSO_4_. The result showed that, compared with that in the original ZE9 strain, the productions of epothilones in YL1616 and YL1624 were all significantly increased (*p* < 0.01). However, compared with that in YL1616, the production of epothilones was not significantly changed in YL1624 grown with 25 μM copper concentration (Fig. 5a). With the copper concentration increased, the production of epothilone B in YL1624 was significantly higher than that in YL1616 (*p* < 0.05 at 120 μM and *p* < 0.01 at 200 μM). Thus, there was a positive correlation between the epothilone production in YL1624 and the copper concentration in cultures. The RT-qPCR analysis also showed that, compared with that of YL1616, the expressions of most of the seven genes were not significantly changed in the YL1624 mutant grown with 25 μM copper concentration (Fig. 5b). With the copper concentration increased, the expressions of most of the seven genes were significantly increased in YL1624, when compared with that in Yl1616. This result indicated that the expression increase of the mxdCas9-activator fusion is able to improve the target transcriptional efficiency, and consequently increase the production of epothilones.

## Conclusions

Efficient expressions of exotic genes in heterologous hosts are not only important for the yield increase of desired products, but also useful in the discovery of novel compounds [41, 42]. Promoters play a fundamental role for the regulation of gene expressions, and many efforts have been made to optimize promoters [43, 44]. CRISPR/dCas9 system can regulate gene expression without the editing performance on the genome or genes, like the eukaryotic RNAi technique [45, 46]. Here, we described a method using sgRNA to guide mxdCas9-activator to improve the expressions of the exotic epothilone biosynthetic genes in *Myxococcus xanthus*. This study is the first report exploring the CRISPRa technique in *M. xanthus* and the CRISPR/mxdCas9-based activation for the gene cluster expression of secondary metabolites. Our results suggested that efficient activation of the CRISPRa technique depends on the capacity of recruiting RNAP, as well as the DNA binding specification of the activator protein. Strong promoter to improve the expression of mxdCas9-activator is beneficial to increase the biosynthesis further. This method provides the inspiration and experience for the exploration of microbial metabolisms. The CRISPR/mxdCas9 technique might be also used for the programmable repression of gene transcription in *Myxococcus*.

## Methods

### Strains and culture conditions

Strains used in this study are listed in Additional file 5: Table S1. *Escherichia coli* DH5α and DH5α (λpir) were used for routine transformations and sub-cloning. *E. coli* GB05-red, a derivative of DH10B [47], was used in the recombination performance. *E. coli* strains were grown routinely in Luria Broth (LB) medium. When appropriate, kanamycin (40 μg/mL) and tetracycline (10 μg/mL) were added in the medium. *Myxococcus xanthus* strains were grown in CYE medium [10 g/L casitone, 5 g/L yeast extract, 10 mM 3-(*N*-morpholino) propanesulfonic acid (MOPS; pH 7.6) and 4 mM MgSO4] [48]. When appropriate, kanamycin (40 μg/mL), tetracycline (10 μg/mL), and apramycin (25 μg/mL) were added. The growth temperatures were 37 °C for *E. coli* and 30 °C for *M. xanthus*.

### Construction of plasmids and mutants

The plasmids and primers used in this study are provided in Additional file 6: Tables S2 and Additional file 7: S3. Plasmid pSW30-mxCas9 was generated by amplifying codon-optimized *mxcas9* with the primers T7cas9Eco-F/cas9Hin-R, which was inserted into the EcoRI/HindIII site of pSWU30. The mutations of D10A (GAC→GGC) and H840A (CAC→GCC) were performed in *mxcas9* of the pSW30-mxCas9 plasmid. The mutated gene was amplified from the plasmid with primers M840In-F/M10In-R and M10Out-F/M840Out-R. Plasmid pSW30-mxdCas9 was constructed with linear–linear homologous recombination mediated by full length RecET in GB05-dir [47]. The *gfp* reporter gene was inserted into the pSW30-mxdCas9 plasmid by using the RecET linear–linear homologous recombination to generate pSWmxdCas9*gfp*.

Fusion of the activation factors (ω subunit, α subunit, σ54, CarQ) with mxdCas9 was achieved by amplification of pSW30-mxdCas9 with primers exeffector-F/exeffector-R, and amplification of genes encoding activation proteins with primers ωRed-F/ωRed-R, αRed-F/αRed-R, σ54Red-F/σ54Red-R, CarQRed-F/CarQRed-R to create the C-terminal fusions, respectively, followed by Red/ET linear–linear homologous recombination. The genes were all PCR amplified from the *M. xanthus* DK1622 genome template. Plasmid p41sg was used to express sgRNA. The plasmid was constructed by amplifying synthetic *pilA* promoter and tracrRNA sequence with primers gRNABamHI-F/sgRNAKpnI-R and inserting at the BamHI/KpnI site of pZJY41. Plasmids p41sg1 to p41sg5 were constructed by using p41sg as a template to amplify DNA fragment with primers sgRNA1-F to sgRNA5-F/sgRNA-R and linked with themselves.

The plasmid with copper-inducible promoter, pSWcuodCas9-ω, was generated by introducing the Pcuo promoter to the pSWdCas9-ω plasmid through amplification of this plasmid with primers PcuoRed-F/PcuoRed-R and expro-F/expro-R, followed by Red/ET linear–linear homologous recombination of the two products.

All the plasmids above were electroporated into *M. xanthus* DK1622 or ZE9. The related genes following its own promoters were inserted at the *attB* site in the genome, forming the related mutant strains. The mutants were then cultivated on CYE plates containing tetracycline (10 μg/mL) and apramycin (25 μg/mL). Plasmids p41sg1 to p41sg5 were electroporated into the constructed mutants, which were cultivated on CYE plates containing tetracycline (10 μg/mL), apramycin (25 μg/mL), and kanamycin (40 μg/mL). The mutants were identified and verified by PCR amplification and sequencing. Restriction enzymes, DNA ligase, and other DNA enzymes were used according to the manufacturers’ recommendations. All fragments were validated by Sanger 3730 sequencing.

### Fluorescence assay

Green fluorescence protein (GFP) assays were performed in *M. xanthus* DK1622 mutant (YL1610). Fluorescence was measured under a fluorescence microscope. In this experiment, background fluorescence, or auto-fluorescence, was measured using a control strain lacking the GFP reporter (*M. xanthus* DK1622).

### Fermentation and product detection

*M. xanthus* strains containing mxdCas9-activator, plasmids with sgRNA and the complete epothilone gene cluster were grown for 24 h in 50 mL CYE medium supplemented with Apra Tet and Km antibiotics. The cultures were used as seeds to inoculate at a ratio of 2:100 into the CYE medium containing 2% of the XAD-16 resin for the adsorption of epothilone products. After 7 days rotation at the 250 rpm and 30 °C, the mixtures of cells and resin were harvested by centrifugation and extracted with two volume of methanol by shaking at room temperature overnight [26]. After centrifuged for 20 min, an aliquot of 20 μL of the sample was injected into a HPLC system interfaced with a Finnigan MSQ classic quadrupole mass spectrometer (Thermo Finnigan, USA). The analysis was carried out on a Shim-pack MRC-ODS RP column (4.6 mm × 250 mm, 4.60 μm; Shimadzu, Japan) at a temperature of 28 °C with a mobile phase of 60% methanol (HPLC grade) and ddH_2_O at a flow rate of 1.0 mL/min. The production levels of epothilones were averaged from three independent cultivations and extractions.

### RT-qPCR

After harvested, the *M. xanthus* mutant cells were adjusted to the concentration of approximately 1.75 × 10^10^ cells/mL. The cell suspension was washed three times using TPM buffer. RNA was extracted immediately with a BIOZOL total RNA extraction reagent (BioFlux) following the manufacturer’s instructions. Genomic DNA contamination was removed by using DNA eraser supplied in the PrimeScript RT Reagent kit with gDNA Eraser (TaKaRa). The purified RNA extracts were transcribed reversely to cDNA and stored in aliquots at − 80 °C. Quantitative real-time PCR was performed in a total reaction volume of 25 μL, containing 250 nM primers, 12.5 μL of SYBR Premix Ex Taq GC mix (TaKaRa), 10.5 μL of RNase-free water (TaKaRa), and 1 μL of a 10-fold-diluted cDNA template. PCR was conducted in a Roche Light Cycler 480 sequence detection system, following the program: 3 min at 95 °C, followed by 40 cycles of 30 s at 95 °C, 30 s at 55 °C, and 15 s at 72 °C. The *gapA* gene, encoding for glyceraldehyde-3-phosphate dehydrogenase, was used as the normalization signal. Calibration curves of *gapA* and *mxdCas9* were generated from a 10-fold dilution of *M. xanthus* ZE9 genomic DNA. The primer pairs used for each gene are listed in Additional file 7: Table S3.

To examine the significance of differences, the Student’s *t* test was conducted using the SPSS software. The significance and the high significance of differences were set as p < 0.05 and < 0.01, respectively. The biological replicates from three independent experiments were employed to calculate the significance values.

## Additional files

**Additional file 1: Figure S1.** The DNA and amino acid sequences of *cas9* and codon-optimized *mxcas9*. After codon optimizing, the amino acid composition of mxCas9 was as same as Cas9. To fit *M. xanthus*, the Codon Adaptation Index (CAI) value has been optimized from 0.05 to 0.97, and GC content has been adjusted from 35 to 61.4%. The mxdCas9 protein was obtained by site directed mutagenesis of D10A and H840A (signed in red letter).

**Additional file 2: Figure S2.** Construction process of the pSW30-mxdCas9 plasmid.

**Additional file 3: Figure S3.** Construction of the pSWmxdCas9gfp plasmid, as well as the plasmids containing the fused genes for mxdCas9 and activators.

**Additional file 4: Figure S4.** The sequence and expression vector of sgRNA. The promoter of *pilA* gene (shown in orange color) from *M. xanthus* drives transcription of sgRNAs. The sgRNA fragment containing the 20-nt specific targeting sequence (green), the tracrRNA scaffold (blue) and the human U6 terminator (red).

**Additional file 5: Table S1.** Strains used in this study.

**Additional file 6: Table S2.** Plasmids used in this study.

**Additional file 7: Table S3.** Primers used in this study.
