# Supplementary figures and images for: CRISPR/dCas9-mediated transcriptional improvement of the biosynthetic gene cluster for the epothilone production in Myxococcus xanthus
Source: Microb Cell Fact. 2018 Jan 29;17:15. doi: 10.1186/s12934-018-0867-1 (PMC5787926; doi:10.1186/s12934-018-0867-1)

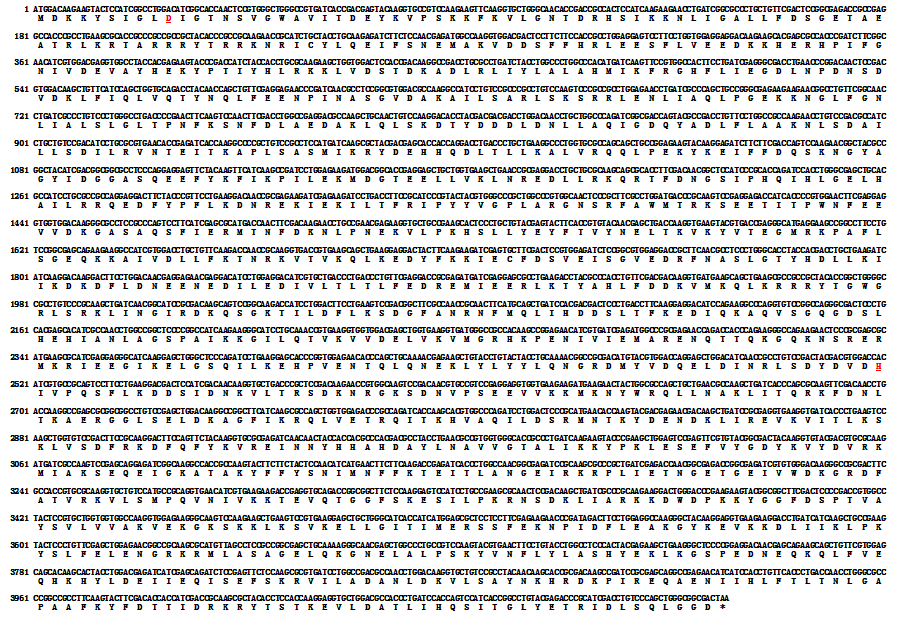

Supplement: Supplementary file 1 — Additional file 1: Figure S1. The DNA and amino acid sequences of cas9 and codon-optimized mxcas9. After codon optimizing, the amino acid composition of mxCas9 was as same as Cas9. To fit M. xanthus, the Codon Adaptation Index (CAI) value has been optimized from 0.05 to 0.97, and GC content has been adjusted from 35 to 61.4%. The mxdCas9 protein was obtained by site directed mutagenesis of D10A and H840A (signed in red letter). [file 12934_2018_867_MOESM1_ESM.bmp]

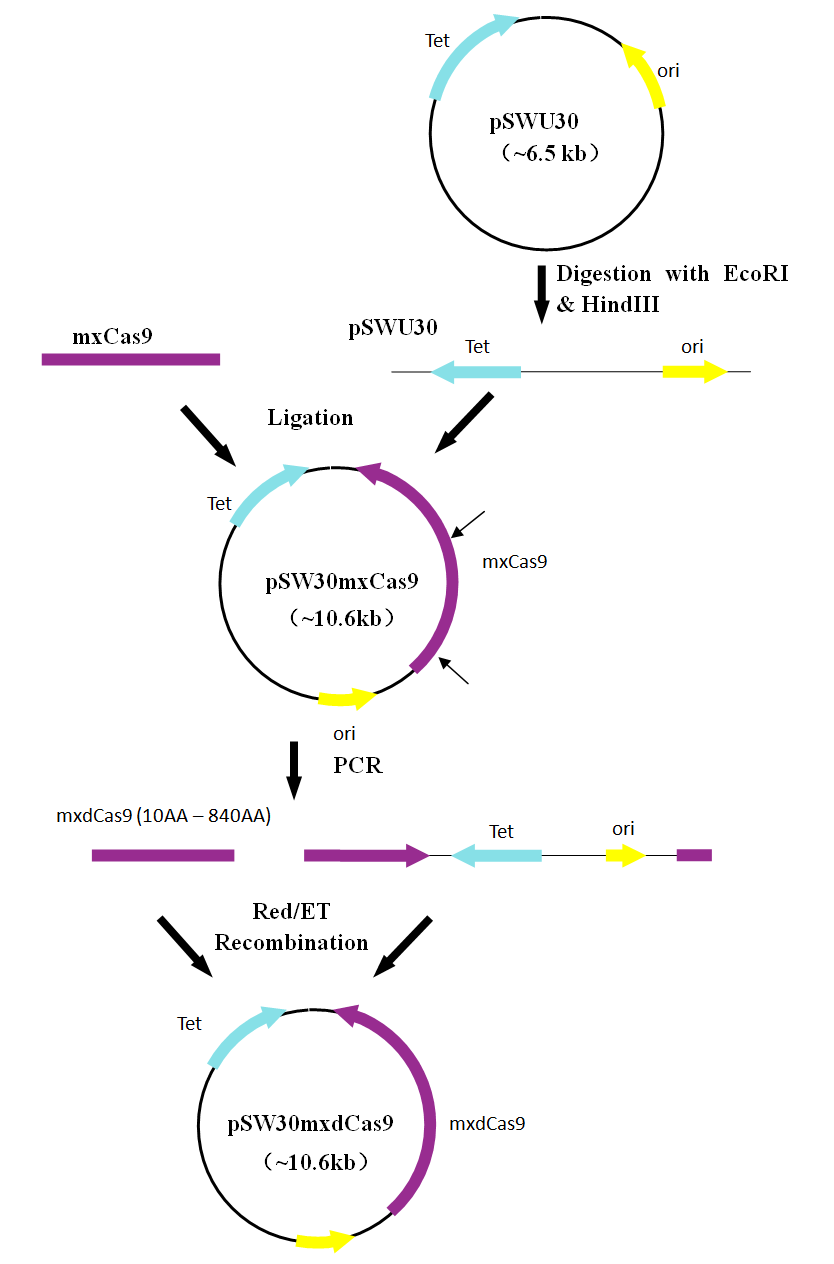

Supplement: Supplementary file 2 — Additional file 2: Figure S2. Construction process of the pSW30-mxdCas9 plasmid. [file 12934_2018_867_MOESM2_ESM.tif]

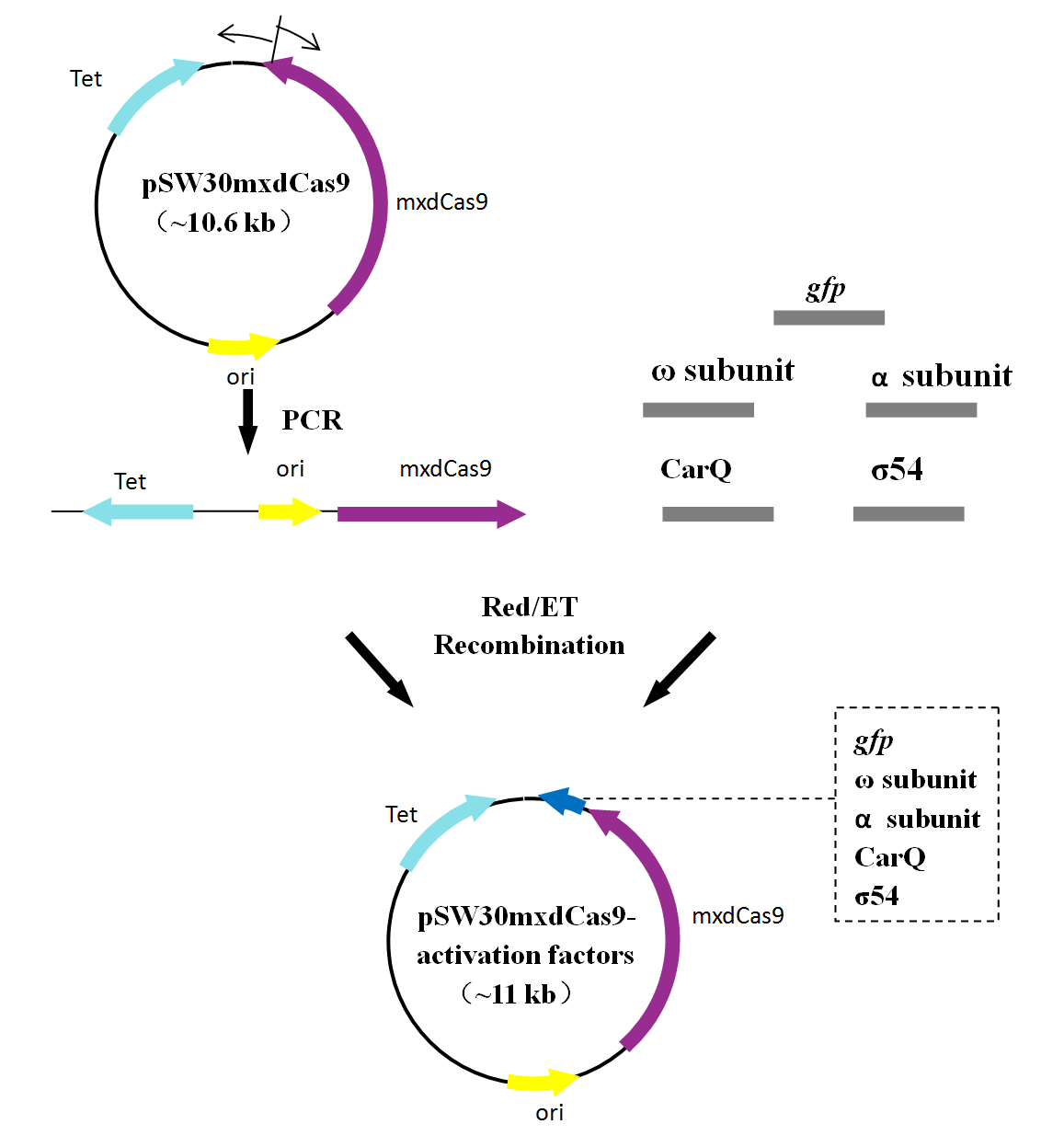

Supplement: Supplementary file 3 — Additional file 3: Figure S3. Construction of the pSWmxdCas9gfp plasmid, as well as the plasmids containing the fused genes for mxdCas9 and activators. [file 12934_2018_867_MOESM3_ESM.tif]

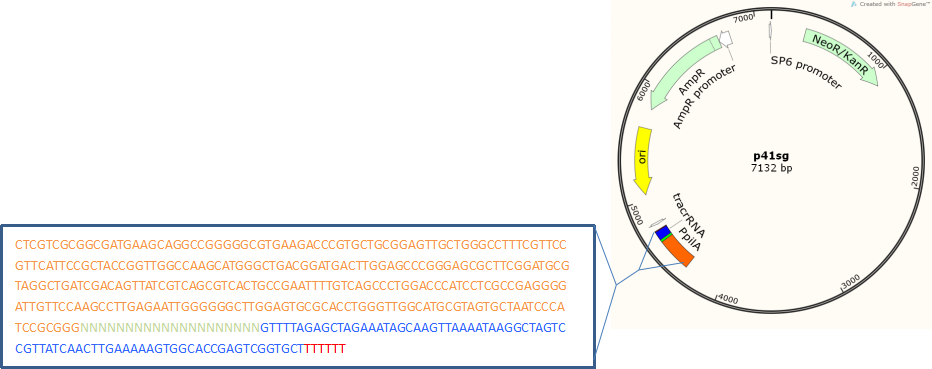

Supplement: Supplementary file 4 — Additional file 4: Figure S4. The sequence and expression vector of sgRNA. The promoter of pilA gene (shown in orange color) from M. xanthus drives transcription of sgRNAs. The sgRNA fragment containing the 20-nt specific targeting sequence (green), the tracrRNA scaffold (blue) and the human U6 terminator (red). [file 12934_2018_867_MOESM4_ESM.bmp]
