# Supplementary material for: CRISPR/dCas9-mediated transcriptional improvement of the biosynthetic gene cluster for the epothilone production in Myxococcus xanthus
Source: Microb Cell Fact. 2018 Jan 29;17:15. doi: 10.1186/s12934-018-0867-1 (PMC5787926; doi:10.1186/s12934-018-0867-1)
Supplement: Supplementary file 5 — Additional file 5: Table S1. Strains used in this study. [file 12934_2018_867_MOESM5_ESM.doc]

| Table S1. Strains used in this study. | | |
| --- | --- | --- |
| **Strains** | **Genotype or description** | **Source or references** |
| *M. xanthus* |  |  |
| DK1622 | Wild-type strains | D. Kaiser, University of Stanford |
| ZE9 | DZ2 with epothilone gene cluster | Zhu et al, 2015 |
| YL1610 | DK1622::pSWmxdCas9gfp | This study |
| YL1611 | ZE9::pSWmxdCas9-Omega | This study |
| YL1612 | ZE9::pSWmxdCas9-Omega( p41sg1) | This study |
| YL1613 | ZE9::pSWmxdCas9-Omega( p41sg2) | This study |
| YL1614 | ZE9::pSWmxdCas9-Omega( p41sg3) | This study |
| YL1615 | ZE9::pSWmxdCas9-Omega( p41sg4) | This study |
| YL1616 | ZE9::pSWmxdCas9-Omega( p41sg5) | This study |
| YL1617 | ZE9::pSWmxdCas9-Alpha | This study |
| YL1618 | ZE9::pSWmxdCas9-Sigma | This study |
| YL1619 | ZE9::pSWmxdCas9-CarQ | This study |
| YL1620 | ZE9::pSWmxdCas9-Alpha( p41sg5) | This study |
| YL1621 | ZE9::pSWmxdCas9-Sigma ( p41sg5) | This study |
| YL1622 | ZE9::pSWmxdCas9-CarQ( p41sg5) | This study |
| YL1623 | ZE9::pSWcuomxdCas9-Om | This study |
| YL1624 | ZE9::pSWcuomxdCas9-Om( p41sg5) | This study |
| *E. coli* |  |  |
| DH5α |  |  |
| DH5α(λpir) | ϕ80 lacZΔM15 ΔlacU169 recA1 endA1 hsdR17 supE44 thi-1 gyrA relA1 λpir | H.B. Kaplan, University of Texas |
| XL1-Blue MR | Δ(mcrA)183Δ(mcrCB-hsdSMR-mrr)173 endA1 supE44 thi-1 recA1gyrA96 relA1 lac | Stratagene |
| GB05dir |  |  |
