# Supplementary material for: CRISPR/dCas9-mediated transcriptional improvement of the biosynthetic gene cluster for the epothilone production in Myxococcus xanthus
Source: Microb Cell Fact. 2018 Jan 29;17:15. doi: 10.1186/s12934-018-0867-1 (PMC5787926; doi:10.1186/s12934-018-0867-1)
Supplement: Supplementary file 6 — Additional file 6: Table S2. Plasmids used in this study. [file 12934_2018_867_MOESM6_ESM.doc]

| Table S2. Plasmids used in this study. | | |
| --- | --- | --- |
| **Plasmids** | **Genotype or description** | **Source or references** |
| pZJY41 | Km^r^ Amp^r^ | Zhao et al，2008 |
| pSWU30 | Site-specific integration vector with Mx8 attP integration site, Tet^r^ | Mignot Tâm, CNRS(Centre national de la recherché scientifique) |
| pSW30-mxCas9 | codon-optimized cas9 gene, with T7A1 promoter sequence, inserted into EcoRI/HindIII sites of pSWU30, Tet^r^ | This study |
| pSW30-mxdCas9 | codon-optimized cas9 gene with （D10A and H840A）with T7A1 promoter sequence, inserted into EcoRI/HindIII sites of pSWU30, Tet^r^ | This study |
| pSWmxdCas9gfp | gfp report gene inserted into the downstream of pSW30-mxdCas9, Tet^r^ | This study |
| pSWmxdCas9-Omega | Omega subunit gene inserted into the downstream of pSW30-mxdCas9, Tet^r^ | This study |
| pSWmxdCas9-Alpha | Alpha subunit gene inserted into the downstream of pSW30-mxdCas9, Tet^r^ | This study |
| pSWmxdCas9-Sigma | sigma54 gene inserted into the downstream of pSW30-mxdCas9, Tet^r^ | This study |
| pSWmxdCas9-CarQ | carQ gene inserted into the downstream of pSW30-mxdCas9, Tet^r^ | This study |
| p41sg1 | sgRNA scaffold with spacer1 inserted into EcoRI/BamHI sites of pZJY41, Km^r^ | This study |
| p41sg2 | sgRNA scaffold with spacer2 inserted into EcoRI/BamHI sites of pZJY41, Km^r^ | This study |
| p41sg3 | sgRNA scaffold with spacer3 inserted into EcoRI/BamHI sites of pZJY41, Km^r^ | This study |
| p41sg4 | sgRNA scaffold with spacer4 inserted into EcoRI/BamHI sites of pZJY41, Km^r^ | This study |
| p41sg5 | sgRNA scaffold with spacer5 inserted into EcoRI/BamHI sites of pZJY41, Km^r^ | This study |
| pSWcuomxdCas9-Om | Chang the T7A1promoter of pSW30-mxdCas9 with copper-inducible promoter Pcuo, Tet^r^ | This study |
