# Supplementary material for: CRISPR/dCas9-mediated transcriptional improvement of the biosynthetic gene cluster for the epothilone production in Myxococcus xanthus
Source: Microb Cell Fact. 2018 Jan 29;17:15. doi: 10.1186/s12934-018-0867-1 (PMC5787926; doi:10.1186/s12934-018-0867-1)
Supplement: Supplementary file 7 — Additional file 7: Table S3. Primers used in this study. [file 12934_2018_867_MOESM7_ESM.doc]

| Table S3. Primers used in this study. | | |
| --- | --- | --- |
| **Primer name** | **Primer sequence (5’-3’)** | **Production** |
| T7cas9Eco-F | GCTCTAGATATCAAAAAGAGTATTGACTTAAAGTCTAACCTATAGGATACTTACAGCCATCGAGAGGATGGATAAGAAATACTCAATAGGCTT | mxCas9 |
| cas9Hin-R | CCCAAGCTTTTAGTCGCCGCCCAGCTGGGAC |
| M840In-F | GGACTGCGGCACGATGGCGTCCACGTCGTAGTCGGACAGGCGGTTGATGTC | mxdCas9 |
| M10In-R | AAGTACTCCATCGGCCTGGCCATCGGCACCAACTCCGTGGGCTGGGCCGTG |
| M10Out-F | GGAGTTGGTGCCGATGGCCAGGCCGATGGAGTACTTCTTGTCCATCCTC | mxdCas9 |
| M840Out-R | GACTACGACGTGGACGCCATCGTGCCGCAGTCCTTCCTGAAGGACG |
| gfpRed-F | GTACGAGACCCGCATCGACCTGTCCCAGCTGGGCGGCGACTCTGGATCAAGTTCTGGTGCACCTGGAATGGTGAGCAAGGGCGCCGAG | gfp reporter gene |
| gfpRed-R | CAGGGTTTTCCCAGTCACGACGTTGTAAAACGACGGCCAGTGCCAAGCTTTCACTTGTACAGCTCATCCATGCC |
| exeffector-F | AAGCTTGGCACTGGCCGTCG | pSW30-mxdCas9 |
| exeffector-R | TTAGTCGCCGCCCAGCTGGGAC |
| OmegaRed-F | TCACCGGCCTGTACGAGACCCGCATCGACCTGTCCCAGCTGGGCGGCGACTCTGGATCAAGTTCTATGGCTCGCGTTACCGTCGAAG | the activation factors ω subunit |
| OmegaRed-R | CAGGGTTTTCCCAGTCACGACGTTGTAAAACGACGGCCAGTGCCAAGCTTCTACTTGGGCTCGTCGCCCGTGTAC |
| alphaRed-F | TGTACGAGACCCGCATCGACCTGTCCCAGCTGGGCGGCGACTCTGGATCAAGTTCTATGGCTGATACGTTCGTTGCG | the activation factors α subunit |
| alphaRed-R | CCCAGTCACGACGTTGTAAAACGACGGCCAGTGCCAAGCTTCTACGCCTTCGGCTGCGC |
| SigmaRed-F | GAGACCCGCATCGACCTGTCCCAGCTGGGCGGCGACTCTGGATCAAGTTCTATGGCGATGGAACTGAAACAAAG | the activation factors σ54 |
| SigmaRed-R | TCACGACGTTGTAAAACGACGGCCAGTGCCAAGCTTTCAGTAGTACCGCTTGCGCTTGC |
| CarQRed-F | TCACCGGCCTGTACGAGACCCGCATCGACCTGTCCCAGCTGGGCGGCGACTCTGGATCAAGTTCTATGCGGAGCCAAACGGACGAAGC | the activation factors CarQ |
| CarQRed-R | CAGGGTTTTCCCAGTCACGACGTTGTAAAACGACGGCCAGTGCCAAGCTTTCATCGCGCCACCTCCAGCTCC |
| sgRNABam-F | CGGGATCCCTCGTCGCGGCGATGAAGCAGG | pilA promoter and tracrRNA sequence |
| sgRNAKpn-R | GGGGTACCATAGCGCCGCTCGCTGTCGTCG |
| sgRNA-R | CCCGCGGATGGGATTAGC | Plasmids p41sg1 to p41sg5 |
| sgRNA1-F | TGGCCCTTTGAGGGGCTGGCGTTTTAGAGCTAGAAATAGC |
| sgRNA2-F | TCGCGCGATATCGTCGACCCGTTTTAGAGCTAGAAATAGC |
| sgRNA3-F | TTACCCTCGGGGAATTGACTGTTTTAGAGCTAGAAATAGC |
| sgRNA4-F | CATGAAAATGGCCCTTTGAGGTTTTAGAGCTAGAAATAGC |
| sgRNA5-F | TCTCCCAGTCAATTCCCCGAGTTTTAGAGCTAGAAATAGC |
| expro-F | GAATTCGTAATCATGGTCATAGCTG | pSWcuomxdCas9-ω |
| expro-R | ATGGACAAGAAGTACTCCATCGG |
| PcuoRed-F | GCGGATAACAATTTCACACAGGAAACAGCTATGACCATGATTACGAATTCGGCCATGAACGGCACTTCACG |
| PcuoRed-R | ACGGAGTTGGTGCCGATGTCCAGGCCGATGGAGTACTTCTTGTCCATCATGAAGCCTCTTCACGAATGGATG |
| gapA-F | GCCCTGGAAGAGCCTGAACG | gapA gene (RT-qPCR) |
| gapA-R | TGGAGACGATGTGGTGCTTGG |
| QA-F1 | GCGTTCCACTCACCGCTCAT | epoA gene (RT-qPCR) |
| QA-R1 | GCCTTCCCGCTCAGATTGCT |
| QP-F2 | GCTCAACATAACGCTCTTCAACC | epoP gene (RT-qPCR) |
| QP-R2 | CTGGACCTCGATACCGCTCA |
| QB-F3 | ATGGAAGAACAAGATTCCTC | epoB gene (RT-qPCR) |
| QB-R3 | CTCGGAGAAGCGCTGCACGG |
| QC-F1 | GAAGATGCGGTGAGGTTGGTGG | epoC gene (RT-qPCR) |
| QC-R1 | TCGGACGCTGCGATGGCTAC |
| QD-F3 | GTGACAGACCGAGAAGGAC | epoD gene (RT-qPCR) |
| QD-R3 | CCACGATGGCGATCGGCTCG |
| QE-F1 | GCACCGTTTGCGTTAGTAGGG | epoE gene (RT-qPCR) |
| QE-R1 | GCTTGGCTATTATGTCGGTCTCC |
| QF-F2 | GGAGCAAGCGAATCAGAGTG | epoF gene (RT-qPCR) |
| QF-R2 | CGTGGTATCGGGTGAGGAC |
